# Influences of summer warming and nutrient availability on *Salix glauca* L. growth in Greenland along an ice to sea gradient

**DOI:** 10.1038/s41598-022-05322-8

**Published:** 2022-02-23

**Authors:** Angela Luisa Prendin, Signe Normand, Marco Carrer, Nanna Bjerregaard Pedersen, Henning Matthiesen, Andreas Westergaard‐Nielsen, Bo Elberling, Urs Albert Treier, Jørgen Hollesen

**Affiliations:** 1 Department of Biology, Ecoinformatics and Biodiversity, Aarhus University, Ny Munkegade 114-116, Building 1540, 8000 Aarhus C, Denmark; 2 Department of Land, Environment, Agriculture and Forestry, University of Padova, Agripolis, Viale dell’Università, 16, 35020 Legnaro, PD Italy; 3 Center for Biodiversity Dynamics (BIOCHANGE), Department of Biology, Aarhus University, Ny Munkegade 114-116, Building 1540, 8000 Aarhus C, Denmark; 4 Arctic Research Center (ARC), Department of Biology, Aarhus University, Ole Worms Allé 1, bldgs. 1130-1134-1135, 8000 Aarhus C, Denmark; 5 Royal Danish Academy, Institute of Conservation, Esplanaden 34, 1263 Copenhagen K, Denmark; 6 Environmental Archaeology and Materials Science, The National Museum of Denmark, IC Modewegsvej, Brede, 2800 Kgs. Lyngby, Denmark; 7 Department of Geosciences and Natural Resource Management, University of Copenhagen, Øster Voldgade 10, 1350 Copenhagen, Denmark; 8 Center for Permafrost (CENPERM), Department of Geoscience and Natural Resource Management, University of Copenhagen, Øster Voldgade 10, 1350 Copenhagen K, Denmark

**Keywords:** Forest ecology, Plant ecology, Environmental impact

## Abstract

The combined effects of climate change and nutrient availability on Arctic vegetation growth are poorly understood. Archaeological sites in the Arctic could represent unique nutrient hotspots for studying the long-term effect of nutrient enrichment. In this study, we analysed a time-series of ring widths of *Salix glauca* L. collected at nine archaeological sites and in their natural surroundings along a climate gradient in the Nuuk fjord region, Southwest Greenland, stretching from the edge of the Greenlandic Ice Sheet in the east to the open sea in the west. We assessed the temperature-growth relationship for the last four decades distinguishing between soils with past anthropogenic nutrient enrichment (PANE) and without (controls). Along the East–West gradient, the inner fjord sites showed a stronger temperature signal compared to the outermost ones. Individuals growing in PANE soils had wider ring widths than individuals growing in the control soils and a stronger climate-growth relation, especially in the inner fjord sites. Thereby, the individuals growing on the archaeological sites seem to have benefited more from the climate warming in recent decades. Our results suggest that higher nutrient availability due to past human activities plays a role in Arctic vegetation growth and should be considered when assessing both the future impact of plants on archaeological sites and the general greening in landscapes with contrasting nutrient availability.

## Introduction

Plant growth in the Arctic region is limited by a cold climate, a short growing season and low nutrient availability. However, in recent decades air temperatures have been rising^1^ and climate models predict that they will continue to increase by another 2–5 °C by 2100^2^. Altered seasonality and improved growing conditions^3–5^ induced by the warming have already led to vegetation change, like increased woody plant growth in the region^6–9^. This shrubification, and the associated greening, is not happening homogeneously across the Arctic^8^ pointing to the fact that variation in temperature is just one of several factors controlling plant growth. Recent studies have shown that different abiotic and biotic factors such as soil water availability^10^, snowpack behaviour^11–13^ and insect outbreaks^14–16^ also influence the vegetation response. The availability of nutrients is another important controlling factor^17–19^. In general, Artic soils are very low in plant available nutrients such as nitrogen (N) and phosphorus (P)^20,21^. However, nutrient availability is expected to increase in conjunction with the on-going warming through extreme atmospheric deposition events^22^ and increased decomposition of soil organic matter^21,23–25^. Fertilization experiments have shown that even small changes in the availability of N and P affect vegetation composition and plant growth^26,27^. However, the interactions between nutrient availability and climate change on shrub growth have mainly been studied over short observational periods^28,29^. Therefore, little is known about the long-term interactions between climate change, nutrient availability, and shrub growth.

Arctic archaeological sites are often recognized in the field based on a distinctly different plant cover compared to surrounding areas^30^. Investigations suggest that archaeological sites contain more soil nutrients than the surrounding natural environment^30–32^. In Greenland, studies show that soils in archaeological sites contain 2–6 times higher levels of P, water extractable nitrate and ammonium compared to the surrounding natural soils^30^. Furthermore, N contains a significant isotopic fingerprint that can be related to past human activities^32^. The profound nutrient enrichment in the archaeological deposits possibly has caused the vegetation composition to differ significantly from the surrounding natural areas with approximately two times more above ground graminoid biomass^30^. Hence, the activities of past cultures have left imprints on arctic environments that have lasted for centuries^32,33^, providing us with a unique opportunity to study the effects of past anthropogenic nutrient enrichment on plant growth during the recent Arctic warming.

**Figure 1:**
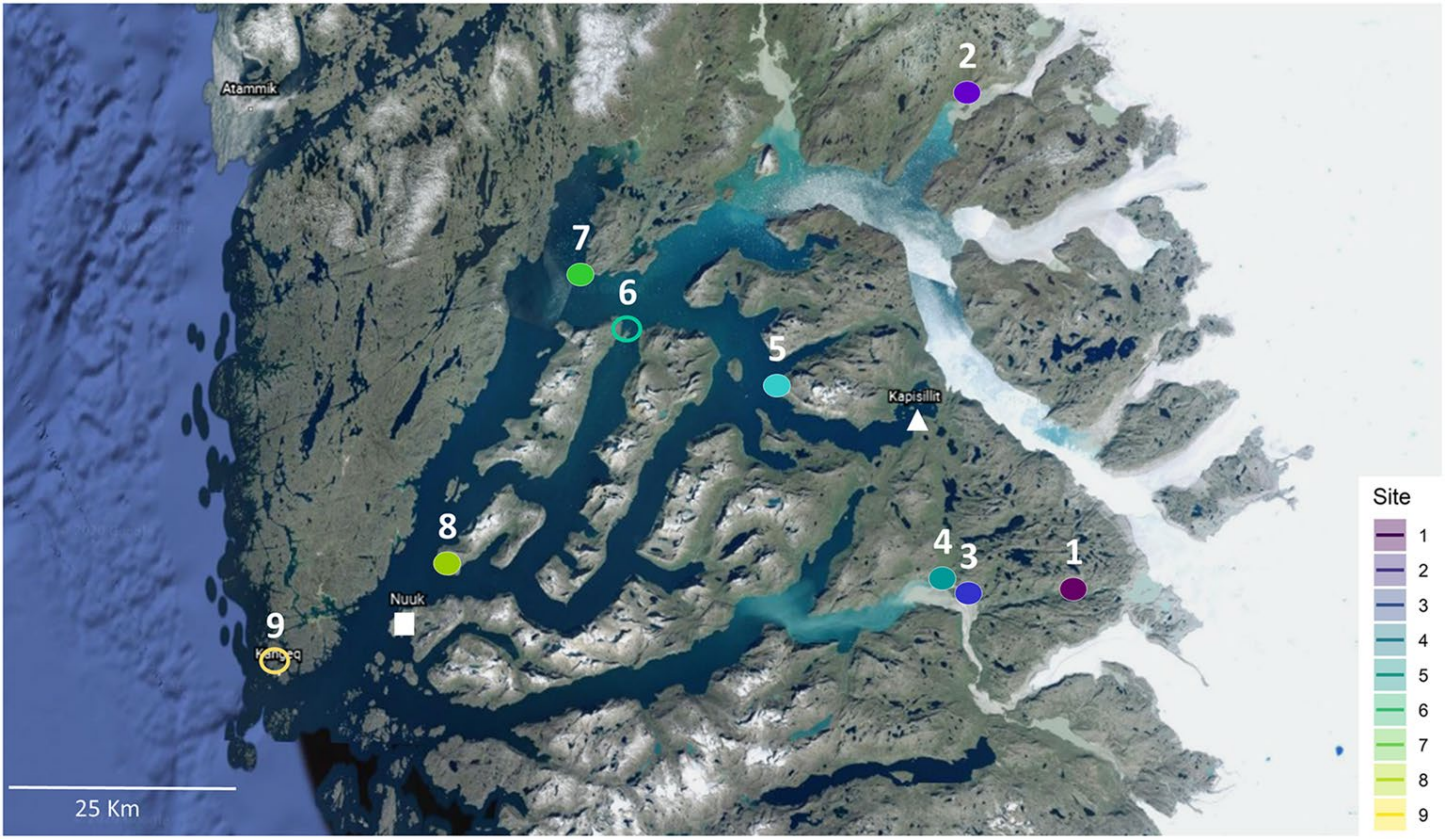
Locations of the study sites in the Nuuk Fjord region in Southwest Greenland. The ice to sea gradient is identified along an East–West transect from the inner fjord to the outer coast. The study sites are: 1: Austmannadal-2 (V53d), 2: Anavik (Ujarassuit), 3: Austmannadal-1 (V52a), 4: Sandnes (Kilaarsarfik), 5: Iffiartarfik, 6: Qoornoq, 7: Nuugaarsuk, 8: Ersaa and 9: Kangeq. The capital Nuuk and the village Kapisillit are identified by a white full square and triangle respectively. Colours (from dark to light) refer to the ice to sea gradient. Filled circles represent sites characterized by past anthropogenic nutrient enrichment (PANE) and control (CONT) samples while empty circles are characterized by PANE only. Figure 1 was generated by Angela Luisa Prendin in Inkscape v1.0.1 (https://inkscape.org/) and it is based on the Nuuk fjord map obtained with Google Earth Pro v7.3 (https://earth.google.com/).

In this study, we investigate the effect of past anthropogenic nutrient enrichment on the long-term temperature-growth response in an area where growth is typically not limited by water. We use dendrochronology to compare annual ring width (i.e., radial growth) of one of the dominant, widely distributed, and long-living deciduous species in the Arctic, Greyleaf willow (*Salix glauca* L.), at nine archaeological sites and in the surroundings areas with low to negligible visible impact from past human disturbance. The study sites are located along an ice to sea climatic gradient in the low-arctic Nuuk fjord region in Southwest Greenland stretching from the inner fjord close to the Inland Ice Sheet and approx. 120 km to the outer coast (East–West gradient) and thus represent a suite of different climatic conditions (from continental to oceanic) (Fig. 1). To our knowledge, this is the first study to explore and quantify the higher sensitivity of warming near archaeological sites where nutrient levels are significantly higher than surrounding natural soil systems while accounting for documented biotic disturbances in the region^16^.

## Results

### Study sites and environmental condition

The nine archaeological sites, distributed along the ice to sea gradient in the Nuuk Fjord (Fig. 1), contain remains from the three main cultures of Greenland: Saqqaq (2,500–800 BC), Dorset (300BC–600AD), and Thule (1,300AD–present), as well as from the Norse Viking Age settlers who inhabited the area from approximately 985–1,350 AD (Table 1).

**Table 1:** Location of sites used for shrub sampling of *Salix glauca* L. in the Nuuk Fjord, Western Greenland and metadata for each site.

Site	Name	Latitude	Longitude	Elevation (m a.s.l.)	T_JJA (°C)	N years	Cultural phases	Nsc/Nsm	Soil	NO_3_ (g/m^2^)	NH_4_ (g/m^2^)	Olsen P (g/m2)
1	Austmannadal − 2 (V53d)	64.2267° N	49.8193° W	230	8.42 ± 0.20	50 (1967–2016)	Norse	12/12	PANE			
								12/12	CONT			
2	Anavik	64.8217° N	50.1491° W	50	8.73 ± 0.22	45 (1972–2016)	Norse	12/12	PANE			
								12/12	CONT			
3	Austmannadal − 1 (V52a)	64.2236° N	50.1219° W	117	7.11 ± 0.19	40 (1977–2016)	Norse	6/6	PANE			
								6/6	CONT			
4	Sandnes (Kilaarsarfik)	64.2438° N	50.1762° W	0–10	8.28 ± 0.22	31 (1986–2916)	Saqqaq, Dorset, Norse	4/4	PANE	0.41 ± 0.09	0.80 ± 0.13	18.21 ± 3.16
								7/7	CONT	0.21 ± 0.05	0.70 ± 0.15	9.77 ± 1.76
5	Iffiartarfik	64.4592° N	50.6455° W	0–10	8.93 ± 0.14	61 (1956–2016)	Norse, Thule, Colonial	12/12	PANE	0.44 ± 0.08	0.80 ± 0.12	12.51 ± 2.4
								12/12	CONT	0.03 ± 0.01	0.41 ± 0.14	4.39 ± 0.70
6	Qoornoq	64.5338° N	51.0857° W	0–10	7.23 ± 0.20	39 (1978–2016)	Norse, Thule, Colonial	9/12	PANE	0.53 ± 0.15	0.85 ± 0.2	29.01 ± 4.93
									CONT	0.04 ± 0.01	0.07 ± 0.02	3.14 ± 0.6
7	Nuugaarsuk	64.6168° N	51.2290° W	0–10	6.934 ± 0.20	45 (1972–2016)	Thule, Colonial	6/6	PANE			
								6/6	CONT			
8	Ersaa	64.2466° N	51.6075° W	0–10	5.84 ± 0.17	80 (1937–2016)	Thule, Colonial	11/12	PANE	0.18 ± 0.03	0.65 ± 0.14	14.85 ± 3.58
								10/12	CONT	0.03 ± 0	0.04 ± 0.02	2.05 ± 0.37
9	Kangeq	64.1072° N	52.0517° W	0–5	3.72 ± 0.15	23 (1994–2016)	Saqqaq, Thule, Colonial	12/12	PANE	0.51 ± 0.15	0.47 ± 0.06	20.89 ± 2.32
									CONT	0.04 ± 0.01	0.16 ± 0.04	2.07 ± 0.43

T_JJA: Average summer temperatures of June, July and August ± standard error (see^16^); N years: number of years measured; Nsc/Nsm: number of samples cross dated/measured; Depth-integrated soil content (0–35 cm) of NO_3_-N NH_4_-N and Olsen P based on soil samples from 3–8, 8–13, 18–23 and 28–33 cm depth^30^, given as mean ± standard error; Note: PANE = Past anthropogenic nutrient enrichment, CONT = Control.

All sites are settlements with ruins and contain well-preserved organic archaeological materials. The sites represent contrasting conditions in terms of age and type of archaeological deposits. On the archaeological deposit, there are no signs of recent deposit of nutrients despite the fact that, from time to time, the sites are visited by locals, hunters and tourists^32^.

The archaeological deposits with clear signs of past human activities (hereafter Past Anthropogenic Nutrient Enriched; PANE) and the surrounding soils with negligible impact from past human activities (hereafter Control; CONT) are characterized by similar environmental conditions (including slope, aspect, geology, and wind exposure). Previous investigations at five of the sites have shown that the content of plant available phosphorus and water extractable nitrate and ammonium is significantly higher in the PANE soils^30,32^(Table 1). Furthermore, measurements of soil water content in soil samples show no marked differences in soil water contents between the PANE and CONT soils within each site (Figure S1).

### Climatic conditions

Overall, the study region is characterized by low arctic vegetation^34^, and in the inner parts of the Nuuk Fjord sporadic permafrost may occur. For the period 1990–2020, the annual mean air temperature in Nuuk is − 1.0 ± 1.3 °C and the annual amount of precipitation is 847 ± 202 mm^35^. At five of the sites (Figure S2), meteorological and environmental monitoring has been carried out as part of a large-scale risk assessment of climate change threats to archaeological sites^36,37^. Data from summer 2017 (June, July and August) show that the study sites are exposed to very different meteorological conditions due to marked regional differences (Figure S2). The inner fjord sites receive less rain compared to the outer coast sites (70 vs 260 mm respectively) (Figure S2). Continuous measurements of soil water content show that these overall precipitation patterns are reflected in soil water contents, with the soil being drier at the sites in the inner fjord (Figure S1). At an official meteorological station located in Nuuk and run by the Danish Meteorological Institute (DMI), the mean summer temperature in 2017 was 6.1 °C compared to an 1990–2020 mean of 6.4 ± 1.2 °C and the sum of precipitation was 287 mm compared to 215 ± 82 mm^35^.

**Figure 2:**
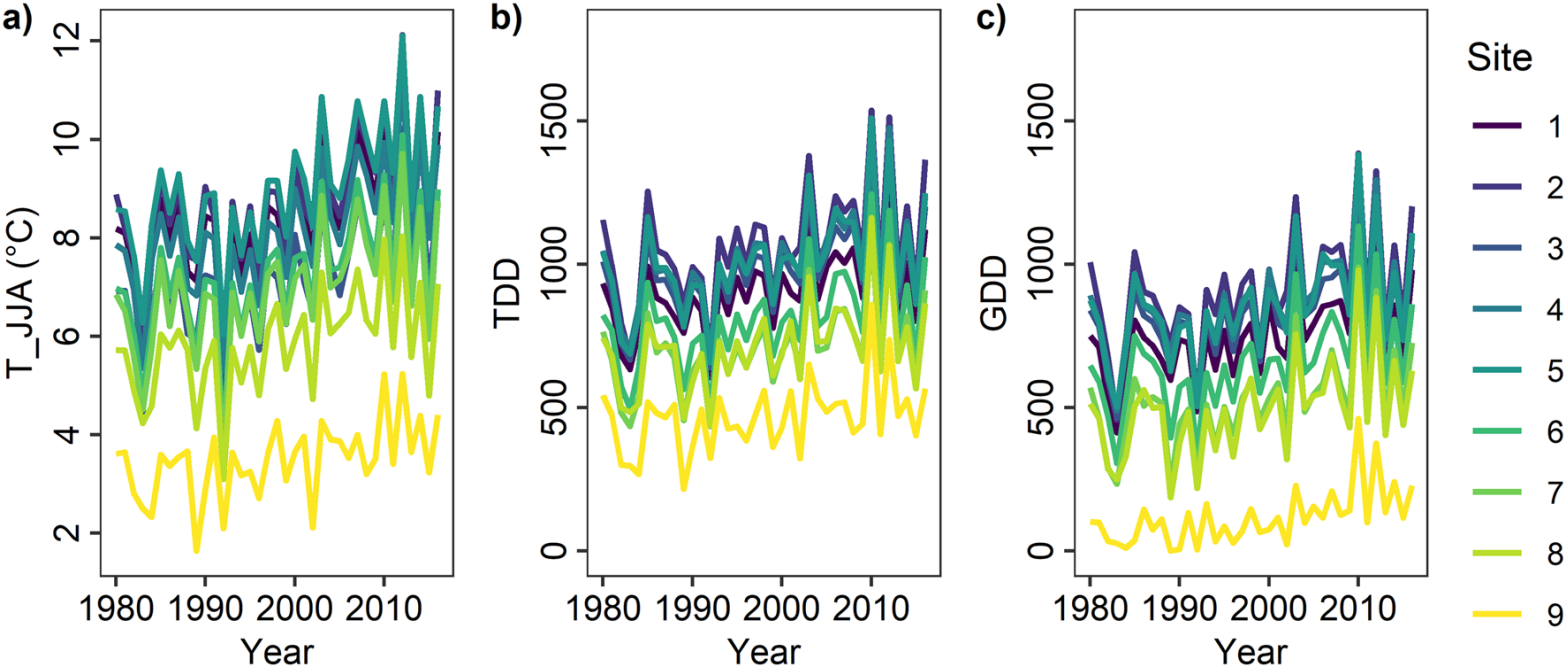
Climate in Nuuk Fjord region. Summer temperature (T_JJA), thawing degree days > 0 °C (TDD) and growing degree days > 5 °C (GDD) time series (period 1980–2016) of the 9 study sites based on the regional climate model MAR 3.7^38^.

Monthly air temperatures for all nine sites were derived from the regional climate model MAR 3.7^38^ to investigate long-term climate–growth relationships (1980–2016) (Fig. 2).

We tested the quality of the modelled air temperatures based on air temperature observations (from 2013–2016) collected at or near three of the sites and almost found a 1:1 relation (*p* < 0.001, R^2^ > 0.95) (Figure S3). The long-term air temperature series confirm the dominating ice-sea climatic gradient in the fjord area with considerable warmer summer temperature (T_JJA) in the inner fjord (Fig. 2). For the period 1980–2016, the average sum of thawing (TDD) and growing degree days (GDD) per year ranged from ~ 1071 ± 30 TDD and ~ 918 ± 30 GDD in the inner fjord (Iffiartarfik and Sandnes) to ~ 470 ± 20 TDD and ~ 117 ± 16 GDD in the outer fjord (Kangeq) (Fig. 2). In addition, summer temperatures show that an overall significant warming (*p* < 0.001) has occurred during the summer period throughout the fjord system since the mid 1990’s (Fig. S4).

### Time series of *Salix glauca* ring width

**Figure 3:**
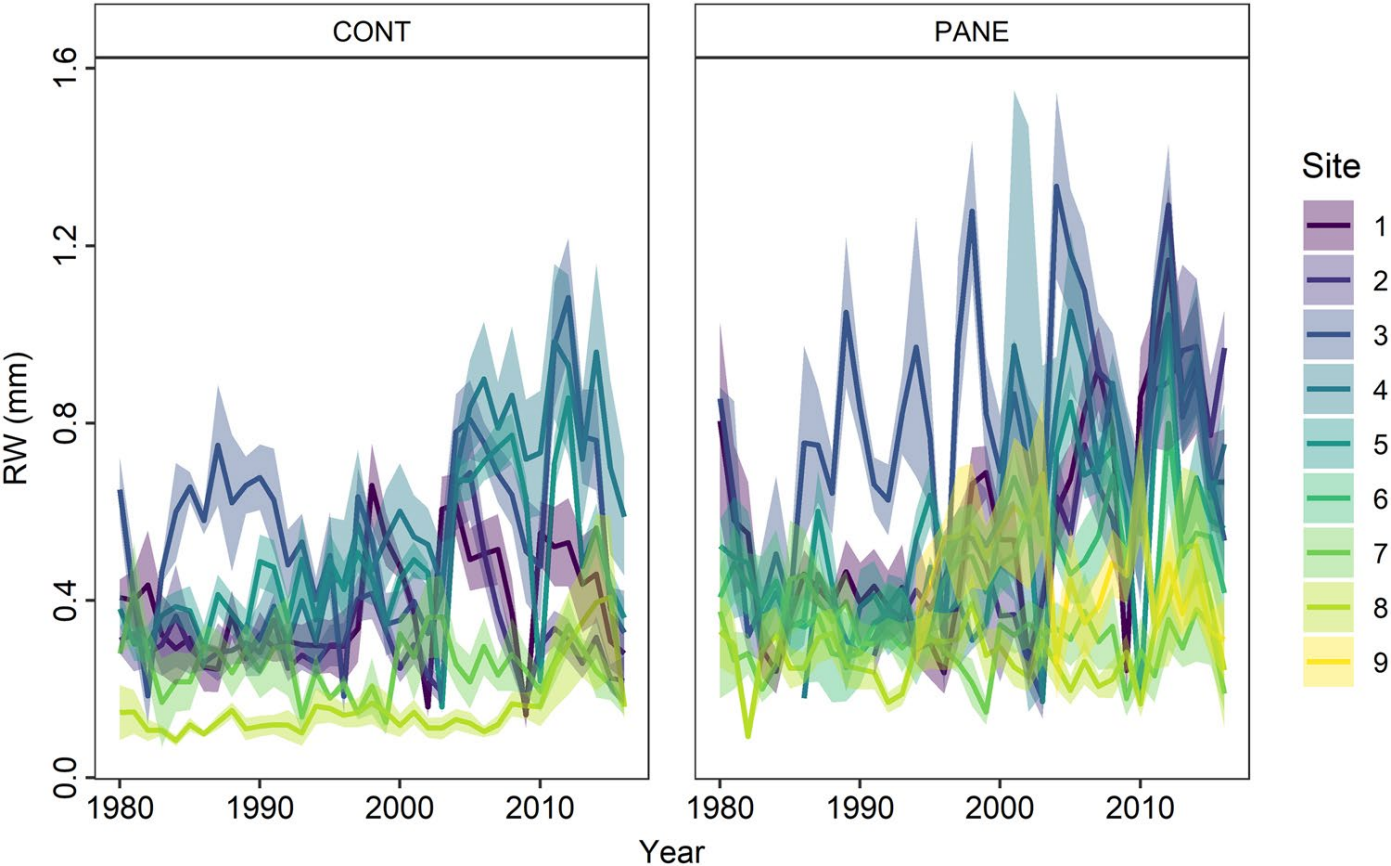
Time series of ring width (mm) of *Salix glauca* L. from nine sites along the Nuuk Fjord (Southwest Greenland). Samples refer to control (CONT) and past anthropogenic nutrient enrichment (PANE) soils. Data are means ± SE. Colours refer to the ice to sea gradient.

Dendrochronological samples of *Salix glauca* L. (hereafter *S. glauca*) collected on archaeological deposits (PANE) and on surrounding soils (CONT) (e.g. Figure S5) ranged from 6 to 12 individuals per sampling site (Table 1). In total nine site chronologies were obtained using 3968 growth rings from 160 dominant and relatively isolated individuals sampled from the PANE and CONT soils. Among all samples, six were excluded from the final chronology as they could not be successfully cross dated due to growth suppressions. At two sites *S. glauca* was only found on the anthropogenically influenced soils (Kangeq) or the surrounding soil was influenced by modern anthropogenic activity (Qoornoq). Hence, for these two sites we built only PANE chronologies. At least five individuals (replicates) covered the 1987–2016 time-period in all site chronologies, while at four sites the chronology went back to 1980 (see Table S1 for chronology descriptive statistics). A clear pattern in ring width (RW) was revealed across the East–West transect, including both PANE and CONT samples. Eastern samples from the inner fjord sites had wider rings and higher common variability (i.e. correlated stronger with each other) than western samples from the outer fjord (Fig. 3 and Figs. S6 and S7).

The mean differences in RW were tested using a paired Student’s t-test to determine whether *S. glauca* growth was significantly different between the PANE and CONT samples. The results show that PANE samples had on average significantly wider rings than CONT (0.051 vs. 0.042 mm, Fig. 3).

### Temperature-controlled growth relationships

**Figure 4:**
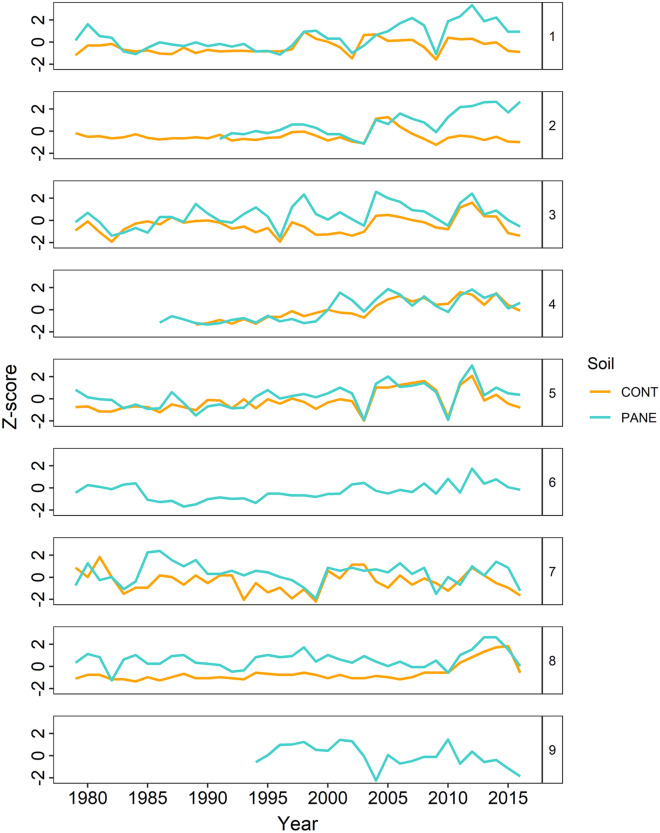
Time series of ring width index (RWI, i.e. Z-scores) of *Salix glauca* L. from nine sites along the Nuuk Fjord (Southwest Greenland). Rings that evidence *Eurois occulta* L. outbreaks are found for the years 2003 and 2010 (Iffiartarfik, Sandnes and Austmannadal-1 (V52a)) and 2002 and 2009 (Austmannadal-2 (V53d)^16^). These rings have been excluded from the analysis. Data represent the site means and colours identify samples from control (CONT) and past anthropogenic nutrient enrichment (PANE) soils.

We investigated the climate-growth associations of *S. glauca* by correlating each of the nine site chronologies (PANE and CONT) (Fig. 4) with the derived time-series of air temperatures using Pearson’s correlation (Table S2).

**Figure 5:**
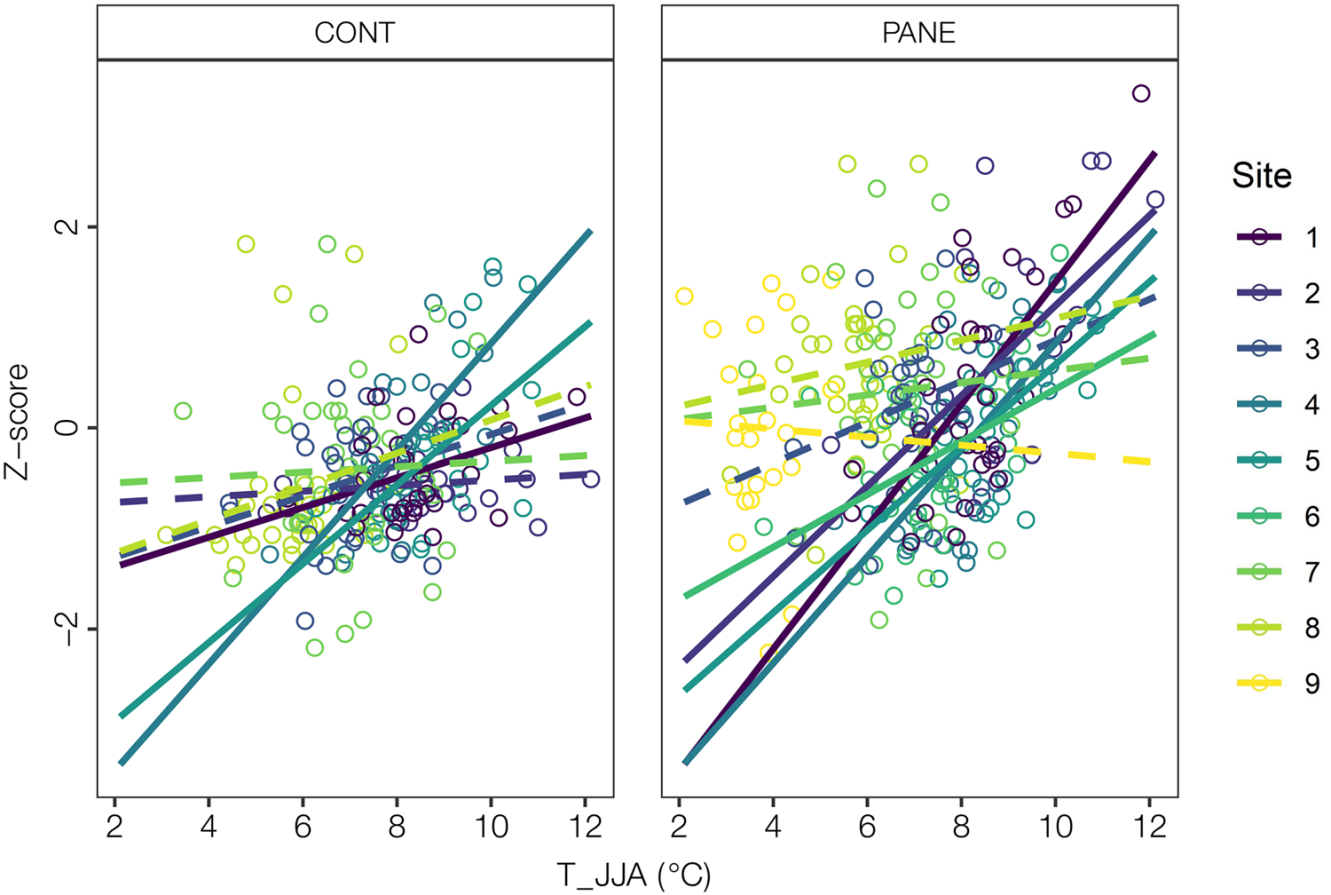
Linear regressions of climate sensitivity of individual growth time series to summer temperatures (T_JJA) for the different sites. Solid and dashed lines indicate significant (*p* < 0.05) and not significant (*p* > 0.05) relationship, respectively. The colour gradient (from dark to light) identifies the different sites located along the ice-sea gradient.

Across all sites, monthly and seasonal mean air temperatures were found to be the most important climate drivers of *S. glauca* growth (Table S2). In particular, the summer temperature (T_JJA) growth relationships were stronger in the inner fjord sites compared to the outer fjord sites (e.g., Pearson’s coefficients range from 0.62 to 0.14, Table S2). Specifically, the explained variance of the linear relationship between ring width and summer temperature ranged from R^2^ = 0.49 to R^2^ = 0.20 following the ice to sea gradient (Fig. 5 and Table S5).

### Effect of nutrient availability on the climate-growth relationship

We used a linear function to describe the climate growth relationship. The linear function, as compared to power, and exponential functions, resulted in the best fit (based on AIC and explained variance) when all sites were considered together (Regional), but also when analysing PANE and CONT sites separately and for the majority of individual sites when excluding the effect of defoliations to avoid disturbance interferences (Table S3).

We assessed the importance of nutrient availability for the climate-growth relationship at the seven sites with pairs of PANE-CONT chronologies using Linear Mixed Effects Models (LMM) and Linear Models (LM). The LMM accounted for the climate-growth associations within sites as well as the growth variability across years (similar to Myers-Smith et al., 2015). The introduction of the nutrient enrichment as a categorical factor in the models (based on the site-specific nutrient measurements see Table 1) contributed to explain the inter-annual variation in growth of *S. glauca* across sites (LMM), as well as within sites (LM). Indeed, when summer temperature and the nutrient enrichment were included in the model (as fixed effects), the variance explained by them (defined as R^2^ marginal) increased from R^2^ = 0.13 to R^2^ = 0.29. When adding the different sites (as random effect) to the model (Regional), the variance explained (defined as R^2^ conditional) increased from R^2^ = 0.20 to R^2^ = 0.36 (Table 2). In addition, when considering CONT and PANE sites separately, the explained variance increased from R^2^ = 0.09 to R^2^ = 0.20 (marginal) and from R^2^ = 0.11 to R^2^ = 0.40 (conditional) (Table2).

**Table 2:** Results of the linear mixed effect (LMM) and Linear (LM) models of the role of summer temperature (T_JJA) and nutrient availability (factor representing (PANE) and (CONT) sample) for the radial growth (RWI, Z-scores) of *Salix glauca* L. (1980–2016). *p < .05, **p < .01 and ***p < .001.

	PANE	Air T_JJA (°C)	Intercept		
**Models across all sites**				R^2^ marginal	R^2^ conditional
Regional		0.23±0.03***	-1.80±0.27***	0.13	0.20
	0.78±0.07***	0.23±0.03***	-2.20±0.26***	0.29	0.36
PANE		0.30±0.05***	-2.00±0.41***	0.20	0.40
CONT		0.14±0.03***	-1.49±0.25***	0.09	0.11
**Models within individual sites**				R^2^	R^2^ adjusted
Austmannadal-2 (V53d)		0.38±0.08***	-3.19±0.71***	0.25	0.24
	0.90±0.18***	0.38±0.07***	-3.63±0.61***	0.47	0.46
Anavik		0.25±0.09**	-2.20±0.78**	0.14	0.12
	1.20±0.20***	0.20±0.07**	-2.27±0.60***	0.50	0.48
Austmannadal-1 (V52a)		**0.18±0.10.**	-1.42±0.67*	0.06	0.04
	0.78±0.17***	0.18±0.08*	-1.81±0.59**	0.32	0.29
Sandnes		0.53±0.10***	-4.47±0.77***	0.41	0.40
	0.01±0.21	0.53±0.10***	-4.47±0.79***	0.41	0.39
Iffiartarfik		0.39±0.07***	-3.54±0.62***	0.34	0.33
	0.42±0.15**	0.40±0.07***	-3.75±0.59***	0.42	0.40
Nuugarsuk		0.04±0.09	-0.31±0.66	0.003	-0.01
	0.73 ±0.22**	0.04±0.09	**-0.68±0.63**	0.14	0.11
Ersaa		0.13±0.11	-0.80±0.67	0.02	0.01
	1.25 ±0.18***	0.14±0.09	-1.42±0.52**	0.42	0.40

The effects were investigated both across all 7 sites (LMM) and for each site individually (LM), excluding the years characterized by insect outbreaks. Numbers indicate the estimates ± SE. R^2^ marginal and R^2^ conditional refer to the variance explained by the LMM models while R^2^ and R^2^ adjusted refer to the variance explained by the LM models.

In addition, the strength of the climate growth associations significantly differs between PANE and CONT in the inner fjord sites, with the nutrient enriched samples showing a larger variance explained compared to the CONT sites (e.g. R^2^ = 0.49 vs. R^2^ = 0.15 in the inner most site) (Fig. 5, Table S4 and Table S5).

### Effect of biotic disturbances

Previous investigations at four of the sites found that annual growth of *S. glauca* is strongly suppressed during outbreaks of the moth *Eurois occulta* L.^16^. Effects of insect outbreaks on annual ring formation were also evident in several of our PANE and CONT samples (Fig. 4 and Figure S6). We accounted for the potential effect of heavy defoliation by the moth by excluding the outbreak year from the chronologies and by including a disturbance factor in the LMM^16^. The explained variation increased to R^2^ = 0.46 when the biotic disturbance factor was also included (Table S4). We found a stronger climate signal in PANE than in CONT samples (Table 2, Table S2, Table S4 and Table S5). When considering the nutrient enrichment factor in a per-site analysis, the explained variation ranged from R^2^ = 0.14 (Nuugarsuk) to R^2^ = 0.53 (Anavik) and even increased to R^2^ = 0.72 (Iffiartarfik) when the biotic disturbance factor was included (Table 2 and Table S4).

## Discussion

An increased greenness (40%) has been seen in the Arctic in the last few decades^39^. The presence of past human activities is well documented all over the Arctic and the legacies of these activities are still visible in the arctic vegetation^33^ with archaeological sites showing increased greenness and distinct species composition with graminoids dominating^30^. The 160 samples of the Greyleaf willow *S. glauca* collected from nine archaeological sites and their natural surroundings located across a marked climatic gradient confirmed a general increase in growth rates during the last decades. In line with previous studies^7,39^, the observed increase in *S. glauca* growth has probably been stimulated by warmer summer temperatures (June, July and August) and longer growing periods (increase in growing degree days). The climate correlation is evident at both PANE and CONT soils and becomes weaker across the climatic gradient from the inner fjord to the outer fjord sites (Figure S7). The weakest correlation was found at the outermost sites (Ersaa and Kangeq) on the west coast with cold, wet and foggy summers. Furthermore, the sites at the outer fjord sites receive significantly more precipitation^35^. High winter precipitation leads to a thicker and longer lasting snow cover, which affects shrub growth^11,12,40^.

A previous study^10^ has also shown that *Salix pulchra* Cham. in mesic habitats may respond positively to a wider range of temperature increase than in dry habitats. We observed a general increase in soil water contents across the ice-sea climate gradient (Figure S1). However, even at the driest sites in the inner fjord the measured soil water contents were well above the threshold for water limitation (25 Vol. %) suggested^10^. Furthermore, the highest growth rates and the strongest correlation with summer temperatures were found at the drier inner fjord sites, suggesting that the observed variation in *S. glauca* growth across the East–West transect is driven especially by variation in temperature. Recent studies have shown that other climate drivers, such as sea ice loss and variability in atmospheric circulation patterns, may affect vegetation patterns at a regional scale^41^. However, the sea in this part of Southwest Greenland is normally not covered by sea ice^35^ and sea ice cover thus might be less important in affecting Greyleaf willow growth rates at our study sites.

Previous studies have defined archaeological sites as nutrient “hot spots” with more above-ground graminoid biomass and different species composition compared to the surrounding areas^30,32^. Our results show that *S. glauca* growth rates were in general higher on the anthropogenic nutrient enriched PANE soils compared to the surrounding CONT soils with negligible impact from past human activities (Figs. 3, 4, Table S3). Furthermore, the strength of the climate-growth relationship was significantly stronger in individuals growing on the PANE soils especially the inner fjord site (Fig. 5). Considering that PANE and CONT samples were collected from individuals facing similar climate and landscape conditions, the difference in growth rates between the two categories appear to be driven mainly by other abiotic or biotic factors varying locally. The higher responsiveness of PANE samples could be, related to the higher nutrient levels due to past human activities. However, it cannot be excluded that a combination of other microclimatic factors also play a role, e.g. a deeper and longer lasting snow cover in and around ruins that may allow for higher water availability during dry periods and less exposure to wind as archaeological sites are generally well sheltered.

Furthermore, archaeological soils are characterized by carbon rich soil layers with high water holding capacities^42^ which are considered important determinants of community composition and biodiversity^43^. However, soil water contents in the PANE and CONT soil during the summer 2017 showed no major differences^30^ and the soil moisture levels were not limiting (see above). It is also well known that biotic interactions, such as insect outbreaks, can cause an instant reduction in productivity and CO_2_ exchange, which is counteracted by a growth release the two following years due to an insect driven increase in nutrient turnover rates^14–16^. Although we found clear evidence of insect outbreaks in several of our PANE and CONT samples, there was no systematic difference between the larvae effect on individuals growing in soils with or without past anthropogenic nutrient enrichment. Previous investigations have shown that the vegetation at the PANE sites is dominated by graminoids and horsetails rather than shrubs^30,32^. Therefore, once established reduced intraspecific and interspecific competition could positively have influenced *S. glauca* radial growth on the PANE sites. However, competition from graminoids has been suggested to affect cover of shrubs in the Nuuk fiord negatively^44^. It is not possible to quantify how these different factors have affected growth rates during the past 3–4 decades, but potentially all factors may have contributed to improve growing conditions of shrubs at the limits of their geographical distribution and in particular at the PANE sites. Nevertheless, although we cannot exclude the effect of microclimatic conditions, we consider nutrient enrichment (seen as higher content of nitrate, ammonium and Olsen phosphorus) in human influenced areas^30^ as the main explanation for the favourable growing conditions of *S. glauca* at the PANE sites.

Climate change and warmer soil temperatures are expected to increase the soil nutrient availability in the Arctic due to accelerated carbon and nitrogen cycling^45,46^. Manipulation experiments with nitrogen and phosphorus have resulted in changes in carbon cycling and vegetation characteristics^47^. Specifically, *Salix* species have increased nitrogen and phosphorus availability with clear positive effects on photosynthesis and biomass production^18,48^. Similar increases in above ground biomass, and photosynthesis have been observed during CO_2_ fertilization experiments in other shrub species^49^ and trees^50^. However, most studies are limited to a single location and reduced time spans^51^. Our study provides valuable new insight on the long-term responses of Arctic shrubs to the effects of fertilization in this temperature-limited ecosystem. Across sites, the *S. glauca* individuals growing on the nutrient enriched archaeological soils were characterized by higher secondary growth due to larger ring width than individuals growing under the same conditions on the nutrient poorer surrounding soils. Although, it cannot be excluded that PANE sites, are slightly more protected than nearby control sites, the PANE individuals seem to benefit more from the recent warming, especially in the inner fjord with a continental climate. This points to the facts that, (i) nutrient availability may have a long-term effect on shrub growth and (ii) spatial variation in nutrient availability in natural environments may lead to a patchy greening response. Our results also highlight the importance of considering effects of past land use when assessing climate-growth relationship for predictions of future vegetation dynamics as well as when using ring width as a proxy for climate reconstruction.

The enhanced growth on the archaeological sites may also have important impacts on the buried archaeological remains, including changes in overgrowth, root development and changes to the water balance^52^. This also means that the site management of archaeological sites in relation to climate change cannot necessarily rely on projections from the natural environment. Our results might suggest that nutrient enriched soils, such as archaeological sites, will experience faster shrubification than natural sites. However, with the current species composition at our study sites, being dominated by graminoids and horsetails rather than shrubs^30,32^, this may not be the case. At the current state of knowledge, we can only speculate about the mechanism, e.g., recruitment of shrubs might be low due to competition at these graminoid dominated lawns. Future studies that could shed light on this paradox could include species competition indices^44^, above and below ground samples (e.g., extent of root, and traits of root growth), water balance over time, effects of abiotic and biotic extreme events (e.g. drought, frost, outbreaks), and surveys or experiments that asses shrub recruitment.

## Conclusion

Almost 6000 archaeological sites are registered in Greenland. In this study, we show the potential of using these archaeological sites to better understand the effect of climate change and nutrient availability on growth of Arctic shrubs. The significant difference in *S. glauca* growth between archaeological sites and natural surrounding areas suggests that the higher nutrient availability due to past human activities plays an important role, even centuries after the sites were abandoned. Our results show the importance of considering not only climate but also past anthropogenic nutrient enrichment, as an important factor that enhances Greyleaf willow growth. Indeed, spatial variations in nutrient availability in natural environments may lead to a patchy response in shrub growth and thus might partly explain the variation greening responses. In addition, our results highlight the importance of considering effects of past land use when assessing climate-growth relationship for predictions of future vegetation dynamics as well as when using ring width as a proxy for climate reconstruction.

## Material and methods

### Study area

The nine archaeological sites in the Nuuk region were visited in August 2016 and 2017 (Fig. 1). The study region is characterized mainly by dry and wet shrub heaths intersected with dry south-facing slopes and smaller fen areas mainly dominated by low shrubs such as *Empetrum nigrum, Salix glauca, Betula nana* and graminoid tundra interspersed by barren bedrock^32,34,53^.

Climate within the Nuuk Fiord varies considerably across space and time. Based on data from an official meteorological station run by DMI (Danish Meteorological Institute), the mean annual temperature in Nuuk from 1990–2020 was − 1.0 ± 1.3 °C and mean annual sum of precipitation was 847 ± 202 mm^35^. Despite an overall warming trend during the summer period (T_JJA) throughout the 1990’s (Fig. 2 and Figure S4), more fluctuating conditions and complex temporal and spatial trends have been observed throughout the fjord system since 2001^54^.

### Dendroecological samples

Dendrochronological samples were collected in relation to the nine archaeological sites. In general, 6–12 individuals of Grayleaf willow (*Salix glauca* L.) were sampled on PANE and CONT soils (Table 1 and Figure S5). Archaeologists from the Greenland National Museum, based on inspections and sub-surface testing, defined the soils influenced by past human activities within each site. The CONT samples were collected from individuals growing under conditions environmentally similar to those of the PANE samples (including slope, aspect, geology, and wind exposure). In total 160 dominant and relatively isolated individuals were sampled by cutting a disc as close as possible to the root collar^55–57^.

At two sites *S. glauca* individuals were only growing on the anthropogenically influenced soils (Kangeq) or the surrounding soil was influenced by modern anthropogenic activity (Qoornoq). For these two sites we built only PANE ring width chronologies (see below). The use of plants in the present study complies with international, national and/or institutional guidelines.

### Soil moisture and nutrients

Measurements of soil water content have been carried out at five of the archaeological sites based on both soil samples and in-situ monitoring^30,36^. The measurements show that water content in the soil (vol%) is similar in the PANE and CONT soils (Table 1). Furthermore, the overall level of soil moisture during summer 2017, which in relation to precipitation represents a “normal year”, is well above the threshold for water limitation of arctic shrub growth^10^ (Figure S1).

Data on plant available phosphorus and water extractable nitrate and ammonium from four of the study sites have previously been reported^30^ (Table 1). Soil nutrient content was determined for samples from the PANE and CONT soils, all collected in vicinity of the dendroecological samples (Figure S5). At all five sites, nutrient content was significantly higher in the anthropogenically influenced soils^30^. Since nutrient content was not available for all sites, we assessed the importance of nutrient availability for the climate-growth relationship of *S. glauca* by comparing samples collected at PANE and CONT soils (Table 2).

### Climate time-series

Climate time-series for all nine sites were derived based on the regional climate model MAR 3.7^38^. MAR 3.7 has been used to model air temperature (3 m above the surface) from 1979 through 2017 (see Figure S3 and Westergaard-Nielsen et al., 2018 for validation of temperatures). The time-series were used to compute the monthly or seasonal climate variables used in this study (Fig. 2 and Table S2). TDD were computed as accumulated daily average temperature above 0 °C and GDD as accumulated daily temperature above 5 °C.

### Shrub-ring chronologies

We followed the standard dendrochronological techniques to prepare the samples of *S. glauca* for ring width (RW) measurement^58,59^. When samples were characterized by very narrow rings, we cut cross sections at 12–15 µm using a rotary microtome (RM2245, Leica, Heidelberg, Germany) and prepared them following a standard protocol^60,61^. Samples with highly visible and wide rings were just sanded. Annual ring widths of each samples were measured and compared, along three to six radii according to stem eccentricity, to detect wedging, missing, and false rings^16^. The ring width measurements were performed to the nearest 0.01 mm with a LINTAB sliding stage micrometre system (Rinn, Heidelberg, Germany). The procedure allowed us to correct any measurement errors and to assign the exact calendar year of formation to each ring. Cross-dating accuracy was verified using the computer program COFECHA^62^.

For each study site, chronologies were created for the PANE and CONT samples separately; at two sites only PANE samples were available. For inter-site chronology comparison and climate association, the RW chronologies (Figure S6) were transformed into Z-scores (standardised to a mean value of 0 and standard deviation of 1)^63,64^. Our final dataset consisted of seven PANE and seven CONT chronologies paired within the seven sites as well as the two PANE-chronologies, each in a different site, where no CONT samples were available (see above).

Descriptive statistics for the chronologies were calculated, in particular: mean sensitivity (MS), an index of the mean relative change between trait-values in consecutive years, assessing the high-frequency variations in the chronologies; mean series inter-correlation (Rbar); and the expressed population signal (EPS)^65,66^ (for details see Table S1).

### Statistical analysis

We investigated the climate-growth relationship of *S. glauca* across the fjord system (Fig. 1) by correlating each of the nine site chronologies (PANE and CONT) with time-series of monthly or seasonal temperature and GDD variables using Pearson’s correlation (Table S2). The mean differences in RW were tested using a paired Student’s t-test to determine whether *S. glauca* growth was significant different between the PANE and CONT samples within each of the seven sites as well as among all nine sites along the ice-to sea gradient.

For all sites together (Regional), for PANE and CONT soils sites separately, as well as for each site individually, we fitted linear, power, and exponential functions to identify which function best described the climate growth relationship (Table S3). The representative models were chosen based on the explained variance and Akaike's Information Criterion (AICc) using the maximum- likelihood method^67^. Then, we assessed the role of nutrient availability for the climate-growth relationship at the seven sites with pairs of PANE-CONT chronologies using Linear Mixed Effects Models (LMM) (when analysing multiple sites together) and linear models (LM) (when analysing the sites individually). The model fits were evaluated by the residual and fitted values^67^. The LMM accounted for the climate-growth associations within sites as well as the growth variability among years^7,68^. The mean site or individual ring width index (Z-score) was considered as response variable. Climate variables were included as fixed effects, together with a factor representing type of soil (PANE or CONT). Site was considered as a random effect when analysing multiple sites together^69^. The annual growth at four of the sites was strongly suppressed during outbreaks of the moth *Eurois occulta* and increased in the two years that followed the outbreak year^16^. We account for the potential effect of heavy defoliation by the moth during outbreak events by removing the outbreak year from the chronologies or by including a disturbance factor in the LMM and LM. The first of these analyses is presented in the main text, while the latter is presented in the supplementary material (Table S4). Finally, we also use linear models to assess the temperature sensitivity of the sub- chronology (PANE and CONT) in each site (Table S5).

We accounted for assumptions of normality and homoscedasticity verifying the normal distribution and random distribution of residuals^67,70^. The optimal models were chosen based on AICc using the maximum likelihood method^67^. Finally, we evaluated the fit of the models by graphical examination of the residual and fitted values^67^.

The ‘lme4’ package in R was used to perform the analyses^71^ and the significance of the fixed effects were tested with F tests^72^ while the variance explained by the fixed and random effects (R^2^ conditional^)^ was calculated for each model using the lmerTest package^73^. All analyses were run with R v 4.1.0.^74^.

## Supplementary Information

Supplementary Information.

## Data Availability

Data associated with this article are deposited in the Dryad Digital Repository 10.5061/dryad.stqjq2c4w.
