# Supplementary material for: Influences of summer warming and nutrient availability on Salix glauca L. growth in Greenland along an ice to sea gradient
Source: Sci Rep. 2022 Feb 23;12:3077. doi: 10.1038/s41598-022-05322-8 (PMC8866482; doi:10.1038/s41598-022-05322-8)
Supplement: Supplementary file 1 — Supplementary Information. [file 41598_2022_5322_MOESM1_ESM.docx]

*Scientific Report*

Supporting information

Influences of summer warming and nutrient availability on *Salix glauca* L. growth in Greenland along an ice to sea gradient

Angela Luisa Prendin^1,2^*, Signe Normand^1,3,4^, Marco Carrer^2^, Nanna Bjerregaard Pedersen^5^, Henning Matthiesen^6^, Andreas Westergaard‐Nielsen^7,8^, Bo Elberling^7,8^, Urs Treier^1,3,4^, Jørgen Hollesen^6^*

^1^Department of Biology, Ecoinformatics and Biodiversity, Aarhus University, Ny Munkegade 114-116, building 1540, 8000 Aarhus C, Denmark.

^2^Department of Land, Environment, Agriculture and Forestry, University of Padova, Agripolis, Viale dell'Università, 16, 35020 Legnaro (PD), Italy.

^3^Center for Biodiversity Dynamics (BIOCHANGE), Department of Biology, Aarhus University, Ny Munkegade 114-116, building 1540, 8000 Aarhus C, Denmark

^4^Arctic Research Center (ARC), Department of Biology, Aarhus University, Ole Worms Allé 1, bldgs. 1130-1134-1135, 8000 Aarhus C, Denmark

^5^Royal Danish Academy, Institute of Conservation, Esplanaden 34, 1263 Copenhagen K, Denmark.

^6^Environmental Archaeology and Materials Science, The National Museum of Denmark, IC Modewegsvej, Brede, 2800 Kgs. Lyngby, Denmark.

^7^Department of Geosciences and natural resource Management, University of Copenhagen, Øster Voldgade 10, 1350 Copenhagen, Denmark

^8^Center for Permafrost (CENPERM), Department of Geoscience and Natural Resource Management, University of Copenhagen, Øster Voldgade 10, 1350 Copenhagen K., Denmark

*corresponding authors:

Aarhus University, Department of Biology, Ecoinformatics and Biodiversity, Ny Munkegade 114-116, building 1540, 8000 Aarhus C, Denmark.

E-mail: angelaluisa.prendin@bio.au.dk

Environmental Archaeology and Materials Science, The National Museum of Denmark, IC Modewegsvej, Brede, 2800 Lyngby, Denmark

E-mail: joergen.hollesen@natmus.dk

**Figure S1 Soil water contents at five of the study sites during the summer 2017.**

**Figure S1:** (a) Soil water content at 0.2 m depth in the PANE soils based on continuous monitoring from 1 June to 31 August 2017. (b) Violin plots of soil water content in samples taken at 0.2 m depth in PANE soils and CONT soils during fieldwork in August 2017, box plots represent the median, the 25^th^ and 75^th^ percentiles, minimum, maximum and outlying points. Colours (from dark to light) refer to the ice to sea gradient.


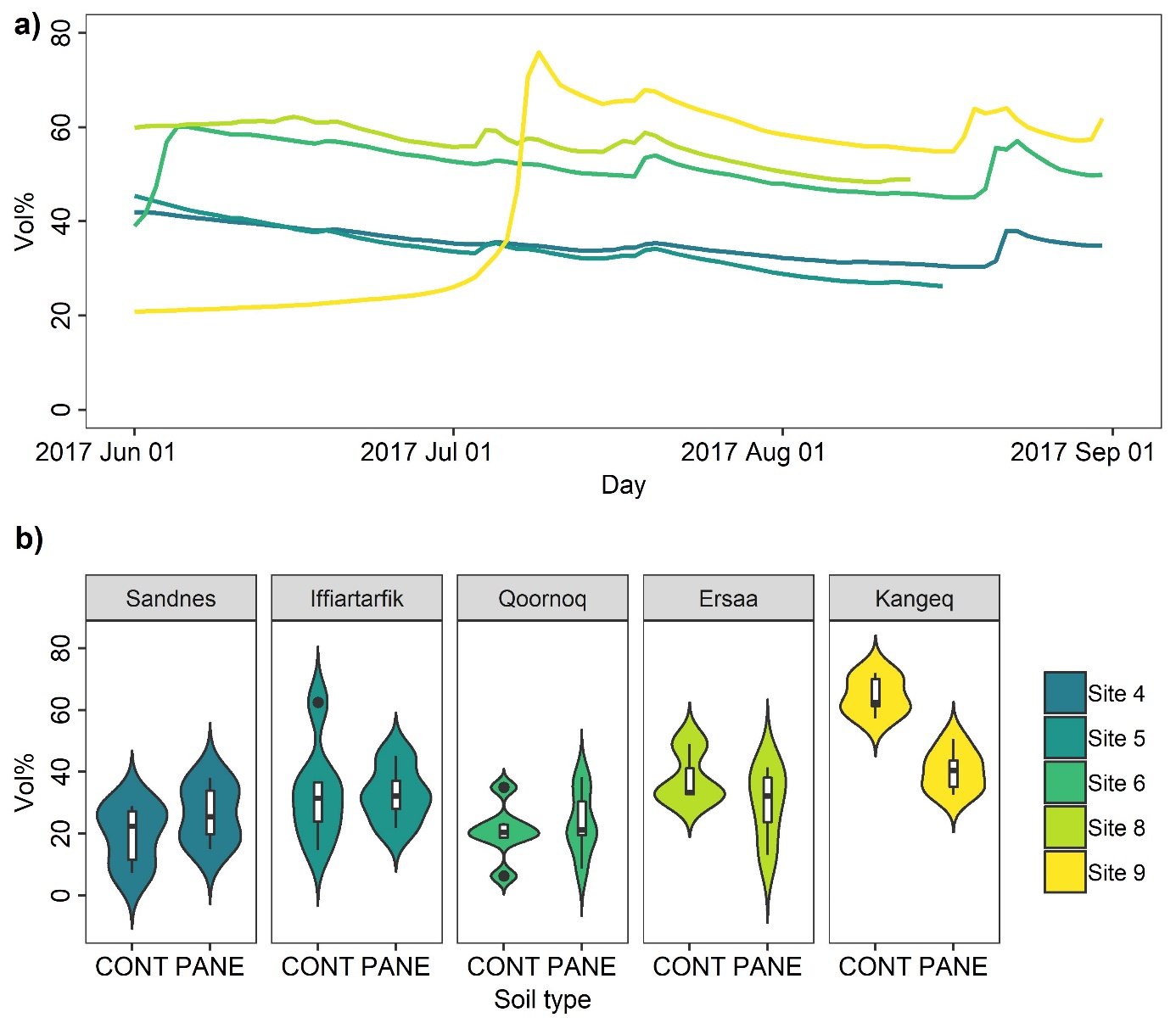


**Figure S2 Climatic observations in the study region from 1 June to 31 August 2017.**

**Figure S2:** (a) mean air temperatures (filled circles) and soil temperatures at 0.1 m depth (empty circles) and 0.4 m depth (triangles). (b) Observed rain precipitation. (c) Sum of precipitation during the growing season June-August (JJA). Colours (from dark to light) refer to the ice to sea gradient.


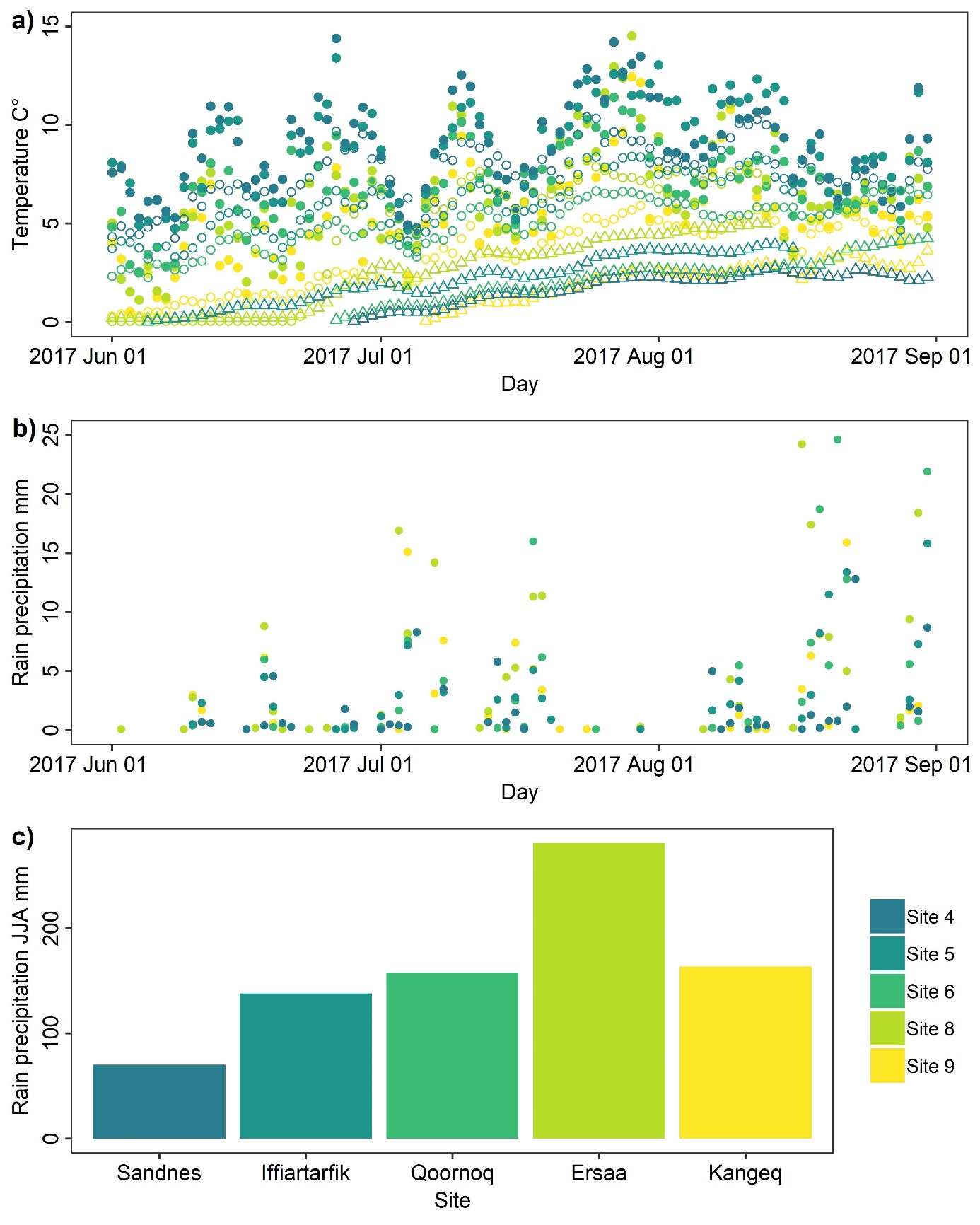


**Figure S3 Relationships between observed air temperatures and modeled air temperatures at three sites.**

**Figure S3:** Monthly averages of the regional climate model MAR (v. 3.7), forced with ERA Interim and observed air temperatures from 2013-2016. For Kangeq and Sandnes (Kilaarsarfik), we used air temperatures measured at the archaeological sites, whereas for Iffiartarfik we used air temperatures from a nearby Danish Meteorological Institute (DMI) station located in Kapisillit. The dashed gray line displays the 1:1 relation. The solid black lines represent linear regressions (p < 0.001, R^2^=0.99, R^2^=0.95 and R^2^=0.98 for Iffiartarfik, Kangeq and Sandnes (Kilaarsarfik) sites, respectively).


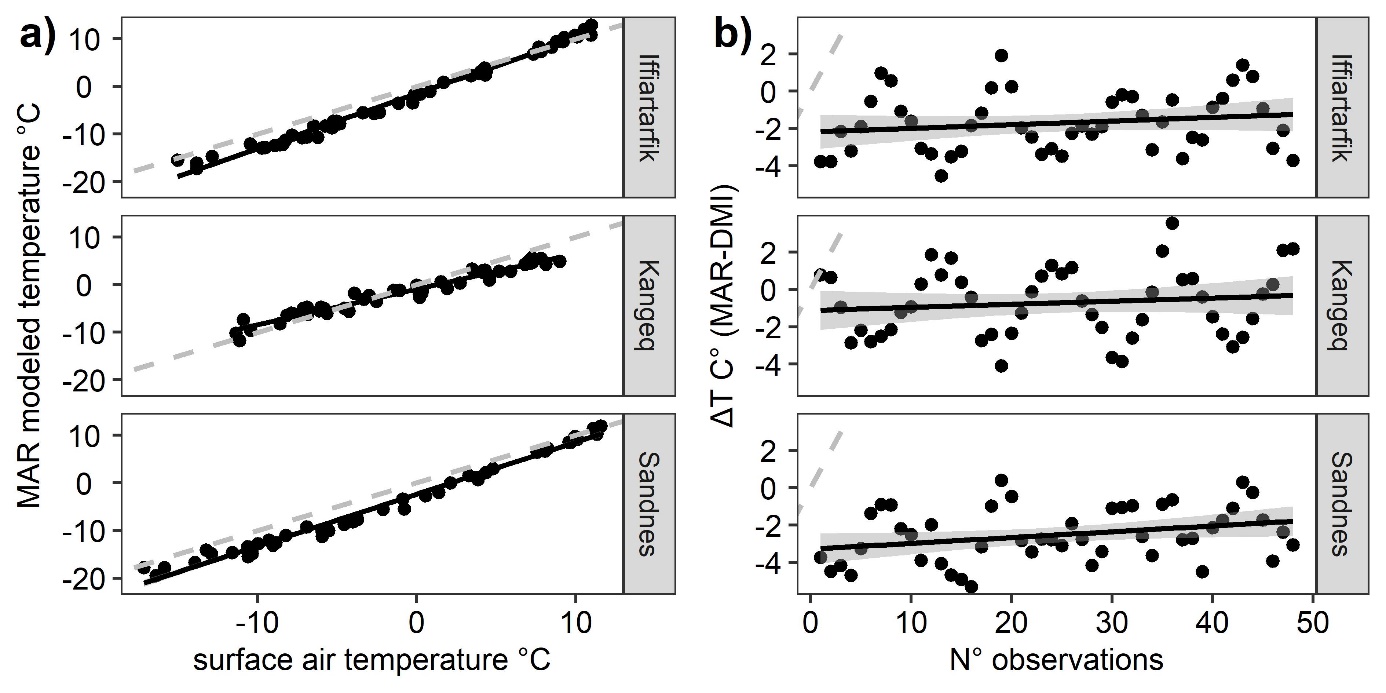


**Figure S4 Warming trend of modelled air temperature in Nuuk fjord.**

**Figure S4:** The positive trend of summer temperatures (T_JJA, represented by empty circles) from 1990-2016 for the nine study sites based on the regional climate model MAR 3.7 (Fettweis et al., 2017). All the sites show a significant trend (solid lines) with the explained variance decreasing following the ice to sea gradient (site 1: p=0.001, R^2^=0.38, site 2: p=0.001, R^2^=0.38, site 3: p=0.001, R^2^=0.36, site 4: p=0.001, R^2^=0.38, site 5: p=0.001, R^2^=0.36, site 6: p=0.003, R^2^=0.30, site 7: p=0.005, R^2^=0.27, site 8: p=0.01, R^2^=0.24 and site 9: p=0.044, R^2^=0.18 performing the ordinary least squares regression). Colours (from dark to light) refers to the ice to sea gradient.


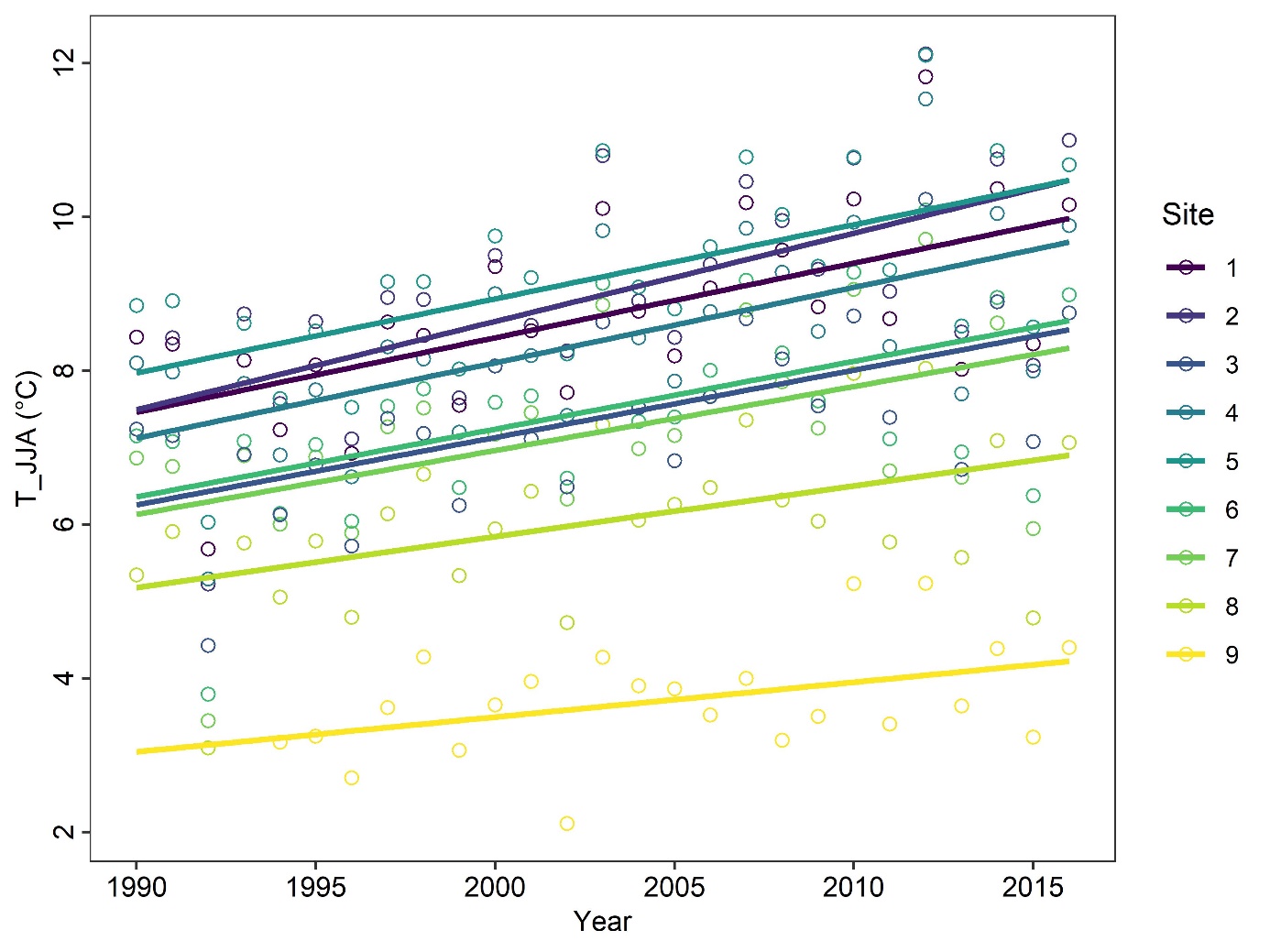


|  |
| --- |
|  |

**Figure S5 The applied sampling design exemplified through the site Iffiartarfik.**

**Figure S5:** Meteorological and environmental monitoring was carried out at one central point (black square). Individuals of *Salix glauca* L. were sampled on archaeological deposits with clear signs of past human activities (PANE) and on surrounding soils with negligible impact from past human activities (CONT). The red and blue dots represent sampling areas where several individuals were collected. Finally, the black circles and triangles show the areas where soil nutrients have been measured by Fenger-Nielsen (2019). The orthomosaic was made by Rasmus Fenger-Nielsen in Agisoft Photoscan 1.4 (Agisoft LLC, St. Petersburg, Russia) (https://www.agisoft.com/) based on RGB images collected with a Sony RX100iii camera mounted on a Tarot 650 quadcopter (see Fenger-Nielsen et al., 2019 for additional details). The map was made by Rasmus Fenger-Nielsen using QGIS 2.14 (https://www.qgis.org/da/site/).


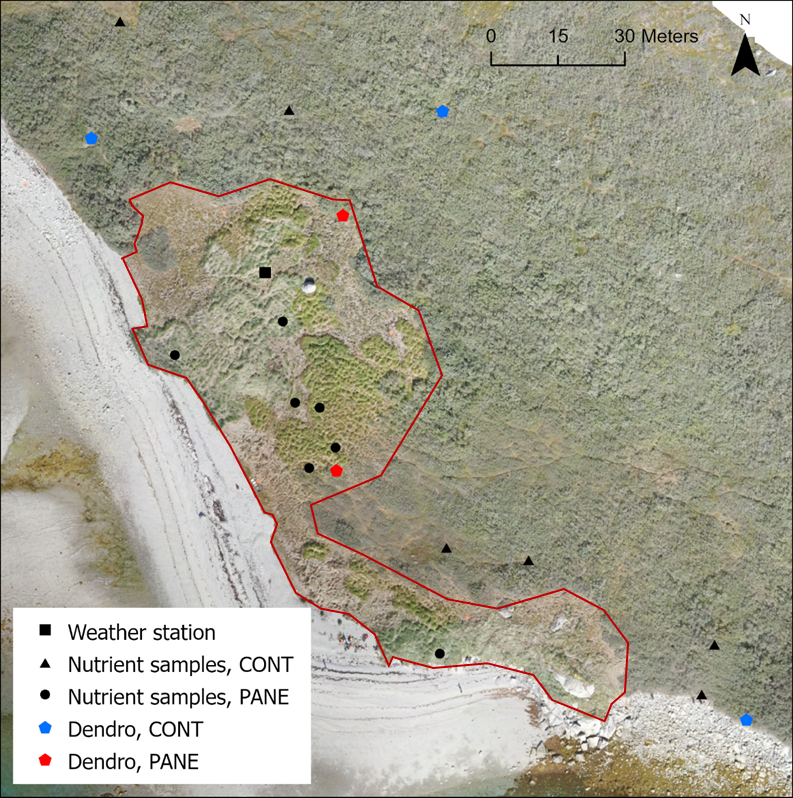


**Figure S6 Time series of ring width (mm) of *Salix glauca* L. from nine sites (from site 1 to 9) along the ice to sea gradient in Nuuk Fjord (Southwest Greenland).**

**Figure S6:** Time series of ring width (RW) of *Salix glauca* L. from nine sites along the Nuuk Fjord (Southwest Greenland) ordered by a) year and b) age. Rings with evidence of *Eurois occulta* L. outbreaks that are clearly visible in 2003, 2010 in Iffiartarfik, Sandnes (Kilaarsarfik) and Austmannadal-1 (V52a) and 2002, 2009 in Austmannadal-2 (V53d) (Prendin et al., 2020) have been excluded from the analysis. Data are a) means ± SE and b) single series. Colours identify samples from control (CONT) and past anthropogenic nutrient enrichment (PANE) soils.

**
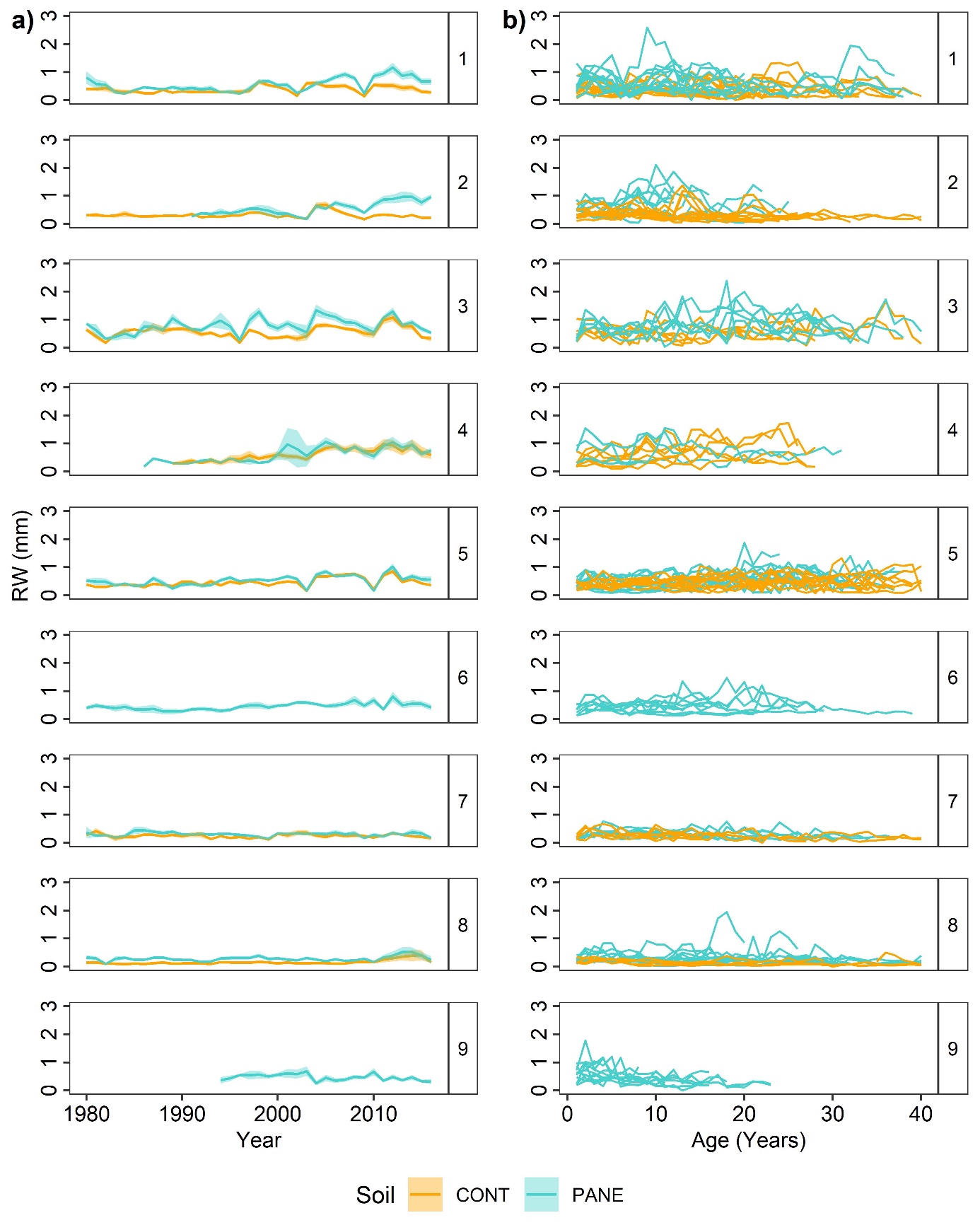
**

**Figure S7 Correlation of ring width (RW) between sites for the common period 1994-2016 (see figure below where sites are ordered following the ice to sea gradient position and soil type).**

**Figure S7:** The correlation coefficient decreases from the innermost sites (characterized by more stable climate condition during the growing season) to the outermost ones. Sites closer to each other show higher correlation coefficient confirming that the position along the fjord significantly affects shrub growth (e.g. site 2, 3 and 4).


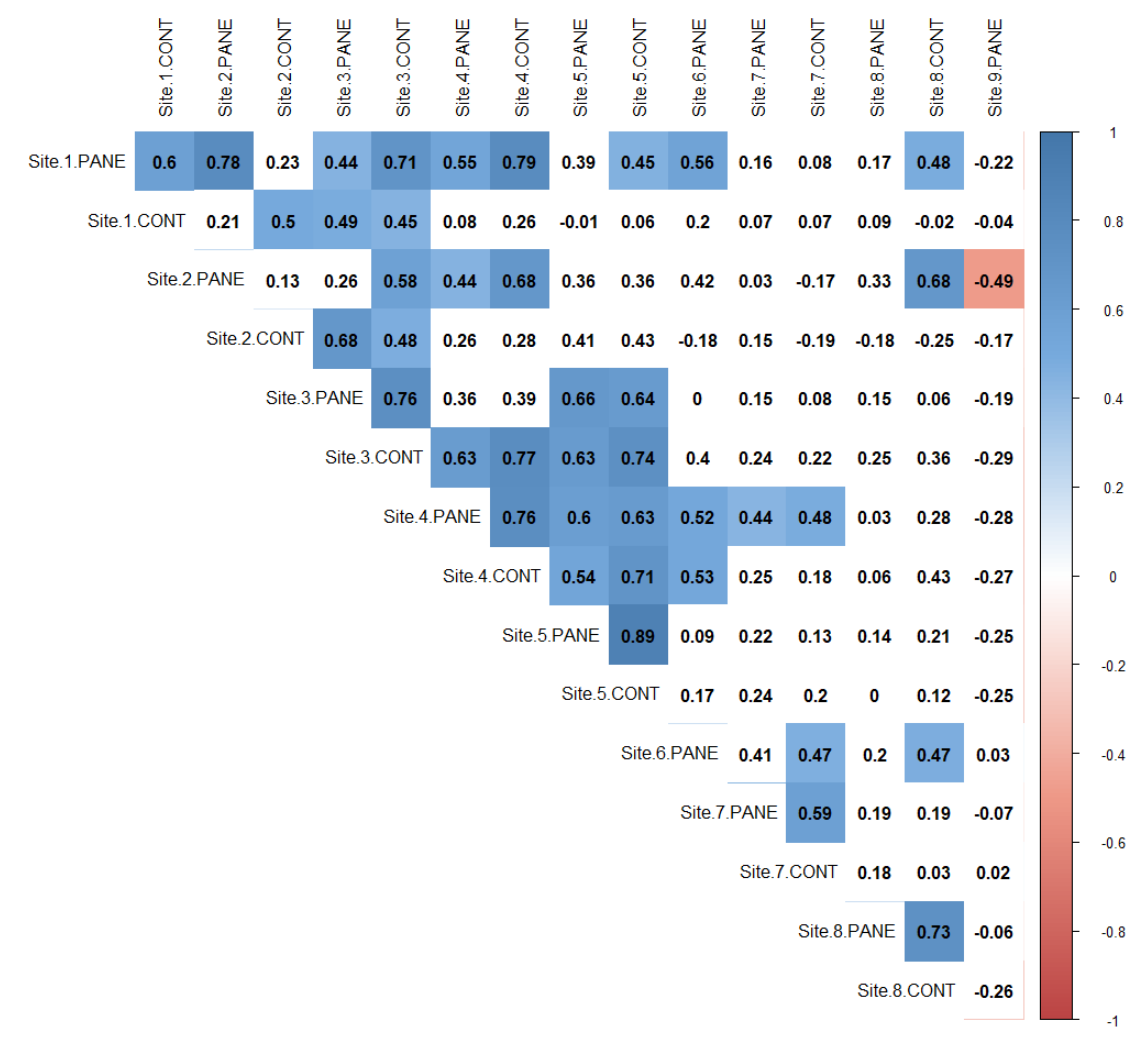


**Table S1 Descriptive statistics of the *Salix glauca* ring width chronologies.**

**Table S1:** Mean ring width (MRW), mean sensitivity (MS), autocorrelation (AC), mean inter series correlation (Rbar) and expressed population signal (EPS). All statistics have been computed on the ring width series for the period from 1994-2016.

| Site | Name | MRW | MS | AC | Rbar | EPS |
| --- | --- | --- | --- | --- | --- | --- |
| 1 | Austmannadalen-2 (V53d) | 0.56 | 0.46 | 0.39 | 0.28 | 0.82 |
| 2 | Anavik | 0.51 | 0.37 | 0.44 | 0.33 | 0.82 |
| 3 | Austmannadalen-1 (V52a) | 0.70 | 0.40 | 0.38 | 0.43 | 0.90 |
| 4 | Sandnes (Kilaarsarfik) | 0.70 | 0.31 | 0.46 | 0.27 | 0.71 |
| 5 | Iffiartarfik | 0.51 | 0.410 | 0.39 | 0.44 | 0.96 |
| 6 | Qoornoq | 0.55 | 0.33 | 0.42 | 0.26 | 0.72 |
| 7 | Nuugarsuk | 0.29 | 0.38 | 0.29 | 0.19 | 0.61 |
| 8 | Ersaa | 0.26 | 0.39 | 0.45 | 0.08 | 0.17 |
| 9 | Kangeq | 0.49 | 0.47 | 0.23 | 0.71 | 0.83 |

**Table S2 Correlation between ring width index (Z-score) and temperature at each site.**

**Table S2**: Pearson’s coefficient of correlation between ring width index (Z-score) and temperature for each month during the growing period individually or combined (T_JJA) and the growing degree days (GDD) above 5°C. Results consider outbreak years (2003, 2010 in Iffiartarfik, Sandnes (Kilaarsarfik) and Austmannadal-1 (V52a) and 2002, 2009 in Austmannadal-2 (V53d) see Prendin et al., 2020) included (Incl.) and excluded (Excl.). *p < 0.05, **p < 0.01 and ***p < 0.001

| **Site ranked** | **Site** name | **Soil**  **type** | **T June** Incl. | **T June** Excl. | **T July** Incl. | **T July** Excl. | **T August** Incl. | **T August** Excl. | **T_JJA** Incl. | **T_JJA** Excl. | **GDD**  Incl. | **GDD** Excl. | **N° Years** | **Period** |
| --- | --- | --- | --- | --- | --- | --- | --- | --- | --- | --- | --- | --- | --- | --- |
| **(ice to sea)** |  |  |  |  |  |  |  |  |  |  |  |  |  |  |
| **1** | **Austmannadal-2 (V53d)** | CONT | **0.376**** | **0.341*** | 0.168 | 0.266 | **0.395***** | 0.275 | **0.418***** | **0.381*** | **0.458***** | **0.407**** | 37(31) | 1980-2016 |
|  |  | PANE | **0.457***** | **0.534***** | **0.454***** | **0.539***** | **0.551***** | **0.601***** | **0.621***** | **0.701***** | **0.630***** | **0.709***** | 37(31) | 1980-2016 |
| **2** | **Anavik** | CONT | 0.115 | 0.162 | --0.121 | 0.148 | -0.075 | -0.014 | -0.006 | 0.134 | -0.060 | 0.103 | 37(31) | 1980-2016 |
|  |  | PANE | 0.276 | 0.384 | **0.426**** | **0.623***** | **0.419*** | **0.614***** | **0.428**** | **0.592***** | **0.394*** | **0.609***** | 26(20) | 1991-2016 |
| **3** | **Austmannadal-1 (V52a)** | CONT | **0.379**** | 0.210 | 0.216 | 0.108 | 0.258 | 0.242 | **0.450*** | 0.260 | **0.376**** | 0.238 | 37(28) | 1980-2016 |
|  |  | PANE | **0.322*** | 0.054 | 0.220 | 0.349 | 0.205 | 0.334 | **0.335*** | 0.293 | **0.321*** | 0.293 | 37(28) | 1980-2016 |
| **4** | **Sandnes (Kilaarsarfik)** | CONT | **0.472**** | **0.502***** | 0.460****** | **0.537***** | **0.596***** | **0.728***** | **0.623***** | **0.630***** | **0.569***** | **0.691***** | 28(22) | 1989-2016 |
|  |  | PANE | **0.395*** | **0.333** | 0.336 | **0.414*** | **0.522***** | **0.519***** | **0.519***** | **0.695***** | **0.439**** | **0.540***** | 31(25) | 1986-2016 |
| **5** | **Iffiartarfik** | CONT | **0.433***** | **0.603***** | **0.433***** | **0.502**** | 0.138 | **0.442**** | **0.405**** | **0.687***** | 0.273 | **0.685***** | 37(31) | 1980-2016 |
|  |  | PANE | **0.412***** | **0.492***** | 0.268 | **0.536***** | 0.167 | **0.347*** | **0.375*** | **0.606***** | 0.231 | **0.592***** | 37(31) | 1980-2016 |
| **6** | **Qoornoq** | PANE | **0.398**** |  | **0.449***** |  | 0.236 |  | **0.449***** |  | **0.525***** |  | 37 | 1980-2016 |
| **7** | **Nuugarsuk** | CONT | -0.023 |  | 0.018 |  | 0.092 |  | 0.028 |  | 0.011 |  | 37 | 1980-2016 |
|  |  | PANE | 0.075 |  | 0.215 |  | 0.151 |  | 0.080 |  | 0.067 |  | 37 | 1980-2016 |
| **8** | **Ersa** | CONT | 0.024 |  | **0.401***** |  | 0.188 |  | 0.219 |  | 0.233 |  | 37 | 1980-2016 |
|  |  | PANE | 0.110 |  | 0.227 |  | 0.053 |  | 0.146 |  | 0.096 |  | 37 | 1980-2016 |
| **9** | **Kangeq** |  | 0.159 |  | -0.074 |  | -0.181 |  | -0.029 |  | 0.090 |  | 23 | 1994-2016 |

**Table S3 Linear, power and negative exponential model selection.**

**Table S3:** Results of the linear, and second order (linear, power and negative exponential) mixed effect models of the role of summer temperature (T_JJA) on growth (ring width index, Z-score) of *Salix glauca* L. (1980-2016). The climate variables were included as fixed effects while site and nutrient availability were considered as a random effect. The effects were investigated both across all sites (PANE and CONT soils) and for each site individually. Numbers indicate the estimates ± SE.

*p < 0.05, **p < 0.01 and ***p < 0.001.

| Model | Disturbance  Incl. Excl. |  | T_JJA (C°) | T_JJA ^2 | Log_10_ (T_JJA ) | Intercept |  | R^2^  marginal | R^2^ conditional |
| --- | --- | --- | --- | --- | --- | --- | --- | --- | --- |
|  |  | Type of model |  |  |  |  | AIC |  |  |
|  | Incl. | Linear | **0.23±0.03***** |  |  | **-0.44±0.09***** | 1319.08 | 0.13 | 0.34 |
|  |  | Power | -0.14±0.17*** | **0.02±0.01**** |  | -0.40±0.70 | 1323.51 | 0.13 | 0.34 |
| Regional |  | Neg. Exp. |  |  | **3.62±0.52***** | **-3.17±0.48***** | 1321.90 | 0.11 | 0.32 |
|  | Excl. | Linear | **0.24±0.03***** |  |  | **-1.86±0.27***** | 1051.66 | 0.14 | 0.40 |
|  |  | Power | **-0.34±0.17*** | **0.04±0.01***** |  | -0.24±0.65 | 10.48.72 | 0.15 | 0.40 |
|  |  | Neg. Exp. |  |  | **0.22±0.03***** | **-3.05±0.46***** | 1057.77 | 0.11 | 0.37 |
| PANE samples | Incl. | Linear | **0.25±0.04***** |  |  | **-1.58±0.34***** | 856.48 | 0.17 | 0.34 |
|  |  | Power | **-0.43±0.20*** | **0.04±0.01***** |  | -0.82±0.75 | 853.94 | 0.17 | 0.30 |
|  |  | Neg. Exp. |  |  |  | **-2.48±0.56***** | 8.61.96 | 0.14 | 0.29 |
| CONT samples | Incl. | Linear | **0.16±0.03***** |  |  | **-1.62±0.26***** | 600.52 | 0.11 | 0.13 |
|  |  | Power | -0.01±0.20 | 0.01±0.01 |  | -1.00±0.81 | 608.70 | 0.11 | 0.13 |
|  |  | Neg. Exp. |  |  | **2.55±0.55***** | **-2.61±0.49***** | 597.24 | 0.09 | 0.11 |
| Austmannadal-2 (V53d) | Incl. | Linear | **0.39±0.07***** |  |  | **-3.28±0.75***** | 192.93 | 0.20 | 0.49 |
|  |  | Power | -0.48±0.63 | 0.05±0.04 |  | -0.38±2.72 | 197.78 | 0.21 | 0.50 |
|  |  | Neg. Exp. |  |  | **7.15±1.41***** | **-6.59±1.37***** | 189.16 | 0.18 | 0.47 |
|  | Excl. | Linear | **0.38±0.07***** |  |  | **-3.18±0.74***** | 152.86 | 0.21 | 0.55 |
|  |  | Power | -0.48±0.58 | 0.05±0.03 |  | 0.43±2.51 | 157.55 | 0.22 | 0.56 |
|  |  | Neg. Exp. |  |  | **6.94±1.37***** | **-6.37±1.33***** | 149.29 | 0.19 | 0.53 |
| Anavik | Incl. | Linear | **0.14±0.07***** |  |  | **-1.13±0.86***** | 168.51 | 0.03 | 0.51 |
|  |  | Power | 0.01±0.57 | 0.01±0.03 |  | -0.56±2.56 | 175.51 | 0.03 | 0.51 |
|  |  | Neg. Exp. |  |  | **2.63±1.40***** | **-2.38±1.44***** | 162.75 | 0.03 | 0.51 |
|  | Excl. | Linear | **0.20±0.07***** |  |  | **-1.68±0.84*** | 125.82 | 0.07 | 0.62 |
|  |  | Power | -0.37±0.51 | 0.03±0.03 |  | 0.73±2.28 | 131.80 | 0.08 | 0.62 |
|  |  | Neg. Exp. |  |  | **3.63±1.31***** | **-3.31±1.37**** | 121.01 | 0.06 | 0.61 |
| Austmannadal-1 (V52a) | Incl. | Linear | **0.28±0.09***** |  |  | **-2.02±0.75***** | 205.79 | 0.09 | 0.39 |
|  |  | Power | 0.27±0.66 | 0.00±0.05 |  | -1.97±2.40 | 212.15 | 0.09 | 0.39 |
|  |  | Neg. Exp. |  |  | **4.48±1.35***** | **-3.79±1.22**** | 200.35 | 0.09 | 0.39 |
|  | Excl. | Linear | **0.18±0.08*** |  |  | **-1.42±0.70*** | 131.20 | 0.05 | 0.43 |
|  |  | Power | 0.56±0.71 | -0.02±0.05 |  | -2.66±2.42 | 137.00 | 0.05 | 0.43 |
|  |  | Neg. Exp. |  |  | **2.81±1.24*** | **-2.50±1.11*** | 125.58 | 0.05 | 0.43 |
| Sandnes  (Kilaarsarfik) | Incl. | Linear | **0.45±0.09***** |  |  | **-3.72±0.72***** | 155.04 | 0.32 | 0.32 |
|  |  | Power | 0.56±0.71 | -0.02±0.05 |  | -1.97±2.40 | 161.39 | 0.32 | 0.32 |
|  |  | Neg. Exp. |  |  | **8.42±1.61***** | **-7.70±1.48***** | 149.37 | 0.32 | 0.32 |
|  | Excl. | Linear | **0.53±0.09***** |  |  | **-4.47±0.77***** | 113.14 | 0.41 | 0.41 |
|  |  | Power | -0.52±0.94 | 0.07±0.06 |  | -1.38±3.73 | 117.72 | 0.42 | 0.42 |
|  |  | Neg. Exp. |  |  | **9.18±1.72***** | **-8.47±1.55***** | 109.17 | 0.38 | 0.38 |
| Iffiartarfik | Incl. | Linear | **0.30±0.09***** |  |  | **-2.70±0.79***** | 215.63 | 0.13 | 0.19 |
|  |  | Power | 0.39±0.82 | -0.01±0.04 |  | -3.08±3.68 | 221.99 | 0.13 | 0.19 |
|  |  | Neg. Exp. |  |  | **6.13±1.72***** | **-5.80±1.64***** | 209.60 | 0.14 | 0.20 |
|  | Excl. | Linear | **0.40±0.07***** |  |  | **-3.54±0.61***** | 127.54 | 0.32 | 0.45 |
|  |  | Power | -0.19±0.69 | 0.03±0.04 |  | -1.05±2.98 | 133.41 | 0.32 | 0.45 |
|  |  | Neg. Exp. |  |  | **7.53±1.30***** | **-7.13±1.23***** | 123.11 | 0.30 | 0.43 |
| Nuugarsuk |  | Linear | 0.04±0.09 |  |  | -0.31±0.71 | 220.97 | 0.003 | 0.22 |
|  |  | Power | -0.16±0.61 | 0.02±0.04 |  | 0.36±2.11 | 227.27 | 0.003 | 0.22 |
|  |  | Neg. Exp. |  |  | 0.55±1.31 | -0.47±1.15 | 215.64 | 0.002 | 0.22 |
| Ersaa |  | Linear | 0.14±0.09 |  |  | -0.80±0.81 | 192.20 | 0.01 | 0.57 |
|  |  | Power | 0.35±0.65 | -0.02±0.06 |  | -1.41±1.97 | 198.03 | 0.02 | 0.57 |
|  |  | Neg. Exp. |  |  | 1.80±1.10 | -1.36±1.04 | 186.93 | 0.02 | 0.57 |

**Table S4 Best linear mixed effect models and linear models.**

Table S4: Results of the linear mixed effect (LMM) and Linear (LM) models of the role of summer temperature (JJA) and nutrient availability (past anthropogenic nutrient enrichment (PANE) and control (CONT) soils) for the growth (Z-score) of *Salix glauca* L.. The effects were investigated both across all seven sites (LMM) and for each site individually (LM) accounting for the effect of insect outbreaks and their carry-over effects (Outbreak disturbance) on growth documented in Prendin et al. (2020). Numbers indicate the estimates ± SE. R^2^ marginal and R^2^ conditional refer to the variance explained by the LMM while R^2^ and R^2^ adjusted refer to the variance explained by the LM.

*p < 0.05, **p < 0.01 and ***p < 0.001.

| Model | PANE | T_JJA (C°) | Outbreak disturbance | | | Intercept | R^2^ marginal | R^2^ conditional |
| --- | --- | --- | --- | --- | --- | --- | --- | --- |
|  |  |  | Outbreak | 1 year after | 2 year after |  |  |  |
|  |  | **0.22±0.03***** |  |  |  | **-1.72±0.27***** | 0.12 | 0.17 |
|  |  | **0.22±0.03***** | **-1.21±0.20***** | **0.88±0.19***** | **1.23±0.19***** | **-1.72±0.26***** | 0.27 | 0.33 |
| Regional | **0.77±0.08***** | **0.22±0.03***** |  |  |  | **-2.10±0.26***** | 0.25 | 0.30 |
|  | **0.77±0.07***** | **0.22±0.03***** | **-1.22±0.18***** | **0.87±0.17***** | **1.22±0.17***** | **-2.10±0.24***** | 0.41 | 0.46 |
| PANE |  | **0.28±0.05***** |  |  |  | **-1.76±0.41***** | 0.16 | 0.30 |
|  |  | **0.28±0.04***** | **-1.51±0.27***** | **0.79±0.26***** | **1.37±0.26***** | **-1.82±0.38***** | 0.33 | 0.48 |
| CONT |  | **0.16±0.03***** |  |  |  | **-1.62±0.27***** | 0.11 | 0.13 |
|  |  | **0.15±0.03***** | **-0.95±0.21***** | **0.93±0.21***** | **1.05±0.21***** | **-1.57±0.25***** | 0.29 | 0.32 |
|  |  |  |  |  |  |  | R^2^ | R^2^ adjusted |
| Austmannadal-2 (V53d) | **0.85±0.16***** | **0.38±0.07***** | **-1.24±0.37**** | -0.03±0.39 | **0.84±0.37*** | **-3.57±0.60***** | 0.53 | 0.50 |
| Anavik | **1.13±0.18***** | **0.17±0.07*** | **-1.19±0.38**** | 0.43±0.38 | **0.84±0.37*** | **-2.01±0.60**** | 0.53 | 0.49 |
| Austmannadal-1 (V52a) | **0.87±0.15***** | **0.22±0.07**** | **-1.05±0.28***** | **1.18±0.28***** | **1.29±0.29***** | **-2.13±0.49***** | 0.61 | 0.58 |
| Sandnes | 0.05±0.19 | **0.44±0.09***** | **-0.75±0.41.** | **1.09±0.38**** | **0.97±0.40*** | **-3.75±0.71***** | 0.51 | 0.46 |
| Iffiartarfik | **0.42±0.13**** | **0.39±0.06***** | **-2.60±0.31***** | **1.17±0.29***** | **1.43±0.30***** | **-3.66±0.52***** | 0.72 | 0.70 |
| Nuugarsuk | **0.73 ±0.22**** | 0.04±0.09 |  |  |  | -0.68±0.63 | 0.14 | 0.12 |
| Ersaa | **1.25 ±0.18***** | 0.14±0.09 |  |  |  | **-1.42±0.52**** | 0.42 | 0.40 |

**Table S5 Temperature sensitivity of *Salix glauca* L. from the nine sites (from site 1 to 9) along the ice to sea gradient in Nuuk Fjord (Southwest Greenland).**

Table S5: Results of the linear models to test summer temperature (T_JJA) sensitivity in past anthropogenic nutrient enrichment (PANE) and control (CONT) soils for the growth (Z-score) of *Salix glauca* L. in the different sites individually. We excluded the years showing the effect of insect outbreaks and their carry-over effects (Outbreak disturbance) on growth according to Prendin et al. (2020). Numbers indicate the estimates ± SE. No significant relations are indicated by NS. *p < 0.05, **p < 0.01 and ***p < 0.001.

| **Model** | **Intercept** | **Slope** | **p** | **R^2^** | **R^2^ adjusted** |
| --- | --- | --- | --- | --- | --- |
| Site 1 CONT | **-1.68±0.56**** | **0.15±0.07*** | 0.035 | 0.15 | 0.12 |
| Site 1 PANE | **-4.63±0.97***** | **0.61±0.12***** | <0.001 | 0.49 | 0.47 |
| Site 2 CONT | **-0.79±0.33*** | 0.02±0.34 | NS | 0.02 | -0.02 |
| Site 2 PANE | **-3.27±1.30*** | **0.45±0.14**** | 0.005 | 0.35 | 0.32 |
| Site 3 CONT | **-1.59±0.78.** | 0.15±0.11 | NS | 0.07 | 0.03 |
| Site 3 PANE | -1.17±0.92 | 0.20±0.13 | NS | 0.09 | 0.05 |
| Site 4 CONT | **-4.47±0.92***** | **0.53±0.11***** | <0.001 | 0.53 | 0.50 |
| Site 4 PANE | **-4.46±1.25**** | **0.53±0.15**** | 0.002 | 0.34 | 0.31 |
| Site 5 CONT | **-3.70±0.84***** | **0.39±0.10***** | <0.001 | 0.37 | 0.35 |
| Site 5 PANE | **-3.48±0.82***** | **0.41±0.09***** | <0.001 | 0.40 | 0.38 |
| Site 6 PANE | **-2.23±0.65**** | **0.26±0.09**** | 0.005 | 0.20 | 0.18 |
| Site 7 CONT | -0.60±0.86 | 0.03±0.12 | NS | 0.00 | -0.03 |
| Site 7 PANE | -0.04±0.90 | 0.07±0.13 | NS | 0.01 | -0.02 |
| Site 8 CONT | **-1.58±0.74*** | 0.17±0.13 | NS | 0.05 | 0.02 |
| Site 8 PANE | -0.01±0.75 | 0.11±0.13 | NS | 0.02 | -0.01 |
| Site 9 PANE | 0.15±1.14 | -0.04±0.30 | NS | 0.00 | -0.05 |
